# Sub-nanoliter, real-time flow monitoring in microfluidic chips using a portable device and smartphone

**DOI:** 10.1038/s41598-018-28983-w

**Published:** 2018-07-13

**Authors:** Yuksel Temiz, Emmanuel Delamarche

**Affiliations:** IBM Research – Zurich, Säumerstrasse 4, 8803 Rüschlikon, Switzerland

## Abstract

The ever-increasing need for portable, easy-to-use, cost-effective, and connected point-of-care diagnostics (POCD) has been one of the main drivers of recent research on lab-on-a-chip (LoC) devices. A majority of these devices use microfluidics to manipulate precisely samples and reagents for bioanalysis. However, filling microfluidic devices with liquid can be prone to failure. For this reason, we have implemented a simple, yet efficient method for monitoring liquid displacement in microfluidic chips using capacitive sensing and a compact (75 mm × 30 mm × 10 mm), low-cost ($60), and battery-powered (10-hour autonomy) device communicating with a smartphone. We demonstrated the concept using a capillary-driven microfluidic chip comprising two equivalent flow paths, each with a total volume of 420 nL. Capacitance measurements from a pair of electrodes patterned longitudinally along the flow paths yielded 17 pL resolution in monitoring liquid displacement at a sampling rate of 1 data/s (~1 nL/min resolution in the flow rate). We characterized the system using human serum, biological buffers, and water, and implemented an algorithm to provide real-time information on flow conditions occurring in a microfluidic chip and interactive guidance to the user.

## Introduction

There is a growing trend to develop portable sensing technologies for point-of-care diagnostics (POCDs), personalized medicine, environmental monitoring, and food safety^1^. Particularly in healthcare, a new generation of POCD systems has recently emerged owing to the rapid adoption of smartphones and their use in LoC devices for sensing, communication, and data processing^2–5^. Majority of these devices strongly benefit from microfluidics due to the possibilities of miniaturizing tests, reducing the consumption of reagents and samples, and decreasing the assay time. There are many varieties of sensing principles^6^, assay formats, and chip fabrication techniques^7^ developed by the community working on microfluidics. One common feature of these techniques is that they require precise manipulation of reagents and samples such as blood, serum, urine, or sweat. Flow is essential to ensure samples and reagents mix efficiently and are transported to specific locations for detection.

A particularly interesting and widely used assay implementation in POCDs is a lateral flow assay, which does not require off-chip sample preparation steps or external fluidic connections^8,9^. Variants of this assay implementation, such as the “one-step” or “self-contained” assays, have been adopted in many other microfluidic devices using passive (e.g. capillary forces), hand-powered, or integrated (e.g. electroosmotic flow) liquid driving mechanisms^10^. Despite their low cost, small size, and ease-of-use advantages compared to conventional clinical analyzers, these assays are prone to failure. Flooding with excess sample, inconsistent flow, unwanted excursion of liquid into signal areas, and subjective interpretation of test results are among the user- and device-related failures that are addressed by the World Health Organization (WHO) for rapid diagnostic tests for Malaria^11^ and by recent review articles^12,13^. A “control signal” is typically present to validate the test, but it does not give a continuous and quantitative feedback on flow, which could be vital for some applications (e.g. detection of cardiac markers in an emergency room, screening of life-threatening infectious diseases in low-resource settings). Therefore, a precise and real-time monitoring of flow is desirable.

Systems with active liquid pumping typically use external flow sensors, close-loop feedbacks, or particle image velocimetry techniques to verify the flow and how much liquid effectively enters a microfluidic device; however, they are bulky and complicated to use for a portable system. Earlier, and still commercially-successful, examples of compact flow sensors have been based on MEMS fabrication and typically use convective heat transfer to estimate flow rates^14^. Some of the more recent flow sensing techniques involve integrated membranes^15^, magnetic nanocomposites^16^, cantilevers^17^, and piezoelectric nanofibers^18^. Although these techniques can achieve good sensitivities (in the range of 1 µL/min) when used with active pumps, they require specific materials and fabrication processes where their combability with portable POCD systems and autonomous flows of liquids have not been demonstrated. In addition, these sensors do not provide a feedback on common failures, such as leakage. Alternatively, flow sensing principles based on electrical detection have been previously reported for microfluidic systems using active pumps^19–22^. These principles can be applied directly to LoC devices that already utilize electrodes for other functionalities, such as detection^23^ and liquid/particle manipulation/trapping^24^. Here, we apply a similar principle to capillary-driven microfluidic systems and demonstrate a simple yet sensitive flow monitoring technique based on a new microfluidic chip architecture using capacitance measurements from pairs of electrodes that are longitudinally patterned along hydrophilic flow paths. This technique also allows an enhanced interaction with the user via a smartphone app, which provides guidance in real-time about the condition of the liquid flowing in the microfluidic chip.

Regarding smartphone-enabled mobile health systems, current research has mostly focused on using phone built-in sensors (e.g. camera, microphone) and connectors (e.g. USB and audio jack) for signal readout and communication. A smartphone opto-mechanical attachment for microtiter-plate reading^25^, an electrochemical analyzer integrated into a smartphone^26^, a dongle using smartphone audio jack^27^, and an image processing algorithm reducing inconsistencies in camera readout^28^ are a few representative examples from many POCD devices using integrated hardware of a smartphone. One issue with these implementations is that the hardware and the form factor of smartphones show significant variations for different models and they change almost in every new generation, limiting the widespread use of these POCD devices. In this paper, we propose a generic and standalone chip peripheral that costs less than $60 and communicates with a smartphone via Bluetooth regardless of the specifications of the smartphone.

## Results

**Figure 1:**
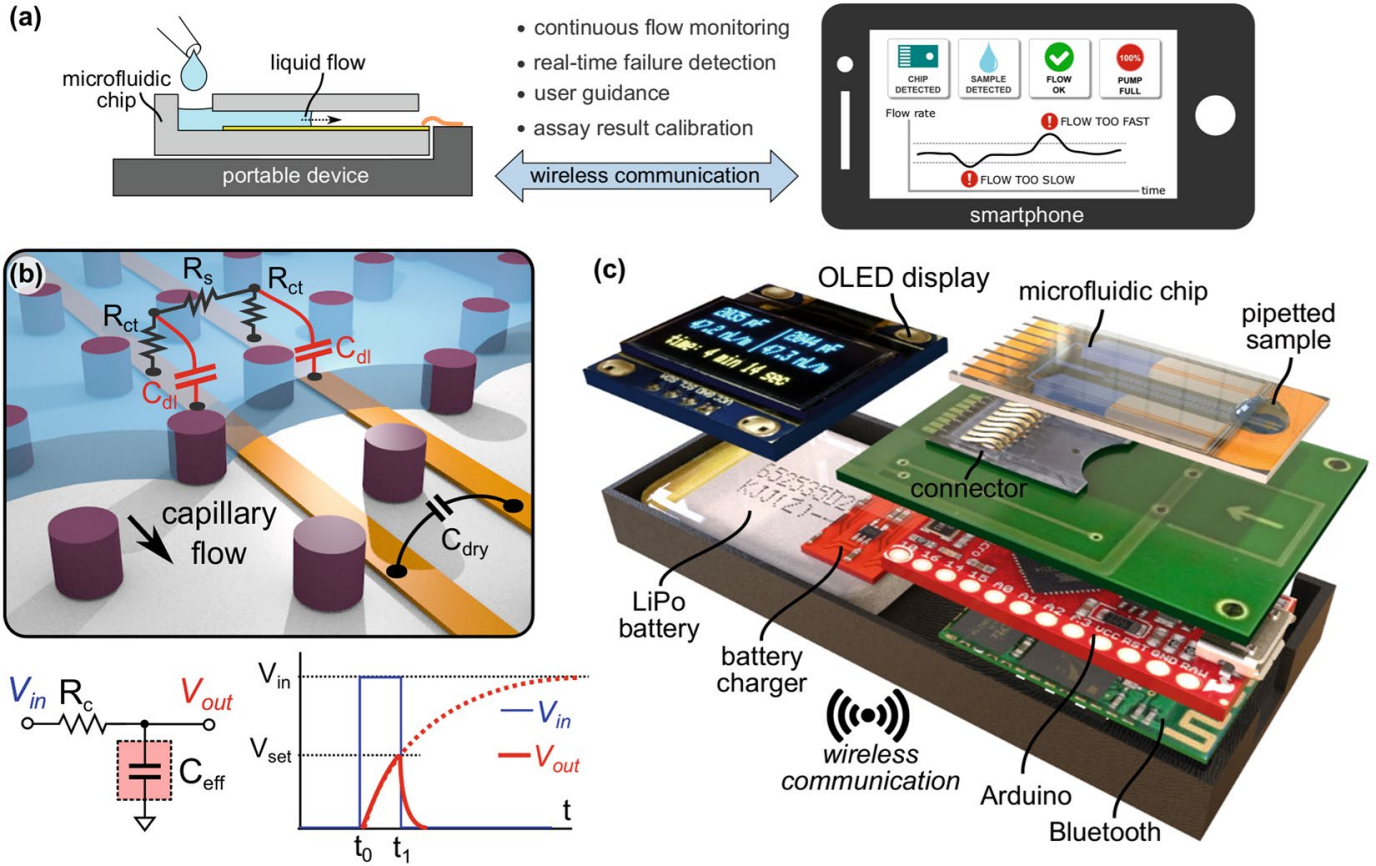
Flow monitoring in microfluidic chips using capacitance measurements and smartphone communication. (**a**) Concept of visualizing flow conditions using a smartphone. (**b**) Illustration of high-precision flow monitoring using capacitance measurements. A liquid gradually filling a microchannel or capillary pump forms a double-layer capacitance (C_dl_) at the electrode interface. The effective capacitance (C_eff_) is then calculated by measuring the time required to charge it to a set value (V_set_) through an external charging resistor (R_c_). (**c**) 3D rendered image of the portable device that can measure capacitances from two independent flow paths, display the results on an OLED display, and send the data to a smartphone via Bluetooth.

An illustration of continuous flow monitoring in microfluidic systems using integrated electrodes and wireless communication is shown in Fig. 1a. The system comprises a microfluidic chip, a portable device to which the chip is connected, and a mobile device (e.g. smartphone or tablet) that continuously communicates with the portable device and displays the position and flow rate of the liquid in the microfluidic chip. The user is warned in real-time regarding potential anomalies such as leakage or blocking of flow due to air bubbles, and is also guided for major steps, such as when to pipette a sample or reagents, and when to read assay results at the end of a test.

### Flow monitoring using capacitance measurements and smartphone communication

The concept shown in Fig. 1b relies on measuring the change in capacitance across two parallel electrodes, which are progressively wetted by a liquid advancing along a microfluidic flow path. In the absence of a liquid, the electrode pair forms a dry-state capacitance (C_dry_), which originates from the fringing lines of the electrical field through the air and substrate (e.g. Si substrate with 200-nm-thick SiO_2_ passivation). When the liquid wets the electrodes, an interface capacitance forms at the electrode-liquid interface of each electrode. Equivalent electrical models of such electrode-electrolyte interfaces have been extensively studied particularly for electrochemical impedance spectroscopy applications^29^. Here, we simplified the measurement principle by assuming that (1) the impedance is dominated by the double-layer capacitance (C_dl_) and (2) the effects of charge-transfer resistance (R_ct_), solution resistance (R_s_), and diffusion (i.e. Warburg impedance) are either negligible or contribute linearly to the measured capacitance. Here, we call the overall capacitance measured from the electrode pair the effective capacitance (C_eff_) and calculate its value by measuring the time required to charge it to a set voltage (V_set_) when an input voltage (V_in_) is applied via an external charging resistor (R_c_). When the initial voltage across the capacitor (V_out_) is zero and V_in_ is applied to C_eff_ through R_c_, V_out_ is calculated as

1 $${V}_{out}={V}_{in}(1-{e}^{-}\frac{({t}_{1}-{t}_{0})}{\tau })$$

where, *τ* is the RC time-constant defined as $\tau ={R}_{c}\cdot {C}_{eff}$. Then, the equation for C_eff_ is derived as

2 $${C}_{eff}=-\,\frac{({t}_{1}-{t}_{0})}{{R}_{c}\cdot ln(1-\frac{Vout}{Vin})}$$

where, the nominator is a measured value (time) and the denominator is a constant.

We designed and implemented compact (dimensions smaller than 75 mm × 30 mm × 10 mm), portable (battery-powered), and low-cost (<$60) peripheral devices that measure C_eff_ from two independent channels based on the equation given above. The device illustrated in Fig. 1c consists of an Arduino microcontroller running with a 3.3 V supply voltage and at 8 MHz clock frequency, an organic light-emitting diode (OLED) display to show the results and warnings, a Bluetooth module to communicate with a smartphone, a 3.7 V lithium-polymer (LiPo) battery, and a microSD connector to interface with the microfluidic chip, where one contact of the electrode pair in the microfluidic chip is grounded and the other contact is connected to the microcontroller (circuit details are given in Supplementary Fig. S1a,b). For applications that require capacitance measurements from multiple flow paths, the ground contacts of each electrode pair can be separated and controlled via the microcontroller to prevent inter-channel cross-talk through the liquid (e.g. 0 V (ground) during the measurement of the selected channel, high-Z (left open) during the measurement of other channels). The capacitance measurement is executed by the microcontroller by applying a 3.3 V (V_in_) voltage difference to the electrode pair via a 10 MΩ R_c_. The voltage across the electrode pair (V_out_) is continuously monitored using the on-chip analog-to-digital convertor (ADC) of the microcontroller while keeping track of the charging time (t_1_ − t_0_) using an internal timer having 1 µs precision. The capacitor is then discharged to 0 V via a 1 K Ω resistor as soon as it reaches 1 V. This voltage limitation prevents undesired electrochemical effects at the surface of wetted electrodes that may occur at higher voltages. The same measurement principle is then applied to a second flow path in the microfluidic chip.

The resolution and the dynamic range of the measurement depend on the charging time (i.e. time-constant) and the characteristics of the ADC and the timer. For instance, it would take respectively 36 µs and 36 ms to charge a 10 pF and a 10 nF capacitance from 0 to 1 V when 3.3 V is applied through a 10 MΩ R_c_. If R_c_ is too high, the measurement would not be fast enough to monitor liquids flowing at high flow rates. If it is too low, the transition from 0 to 1 V would be so fast that it may not be detected precisely using a low-power/low-speed microcontroller. As an example, we used capillary-driven microfluidic chips that generate flow rates of 100 nL/min or less and we measured the capacitance values with a data rate of 10 data/s per channel, then averaged 10 consecutive data points every second and sent them to a smartphone via Bluetooth. The portable devices with a 400 mAh LiPo battery could run up to 10 hours for continuous data communication at this rate (see Supplementary Fig. S1c,d for specifications of other portable devices developed in this work).

### Capillary-driven microfluidic chips with integrated electrodes for flow monitoring

**Figure 2:**
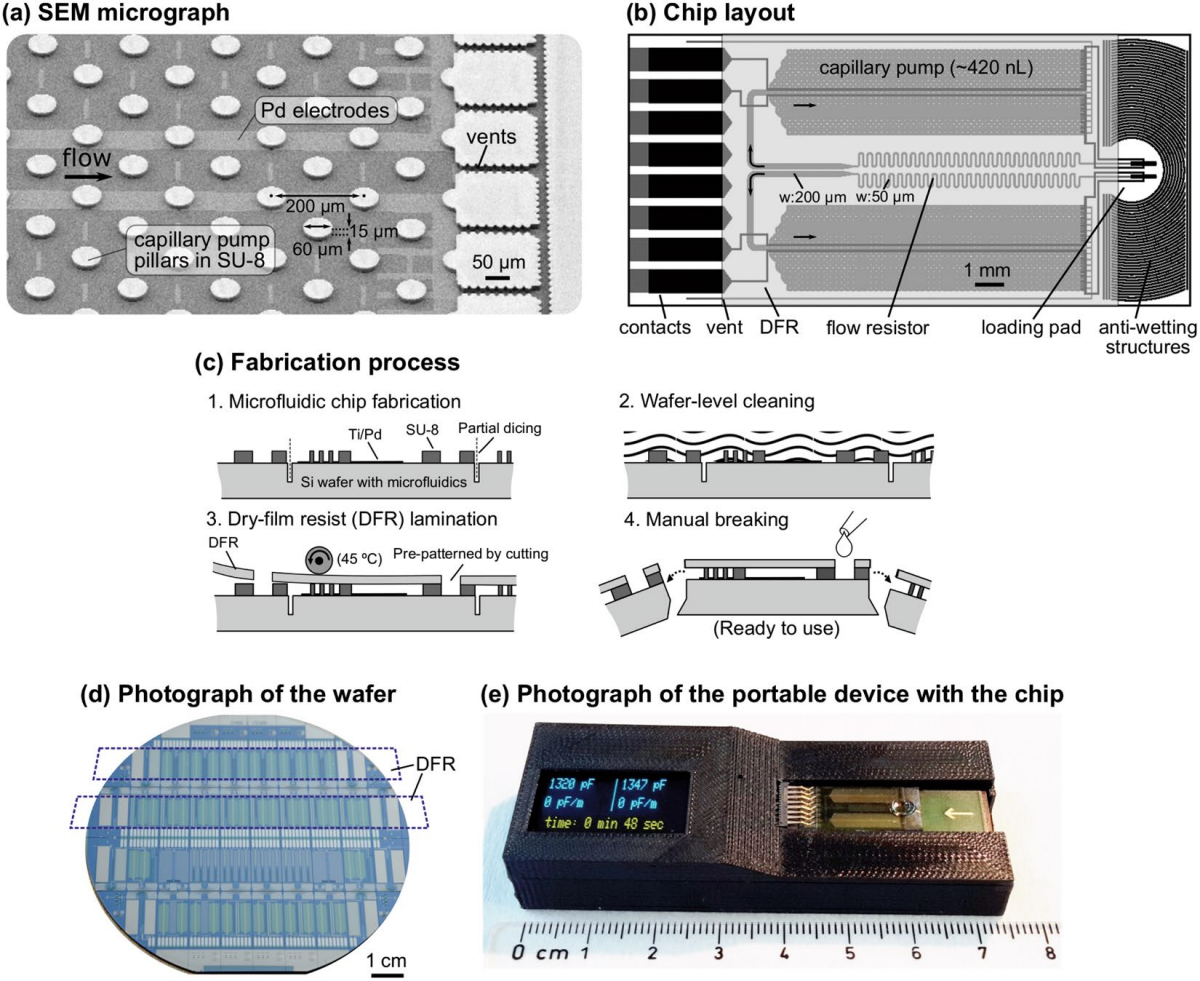
Design and fabrication of capillary-driven microfluidic chips with integrated electrodes. (**a**) SEM image showing a pair of electrodes patterned along a capillary pump. (**b**) Chip layout (9.4 mm × 19.5 mm) comprising two symmetrical microfluidic flow paths, electrodes, and dry-film resist (DFR) for sealing the chip. (**c**) Illustration of the wafer-level fabrication process enabling efficient singulation of “ready-to-use” chips, and (**d**) photograph of the fabricated wafer before chip singulation. The dashed line highlights a rectangular strip of DFR that seals a row of chips without covering their loading pads and electrical contacts. (**e**) Photograph of the portable device after the chip has been inserted and the sample has been pipetted.

The method was illustrated using capillary-driven microfluidic chips having Pd electrodes and SU-8 microfluidic channels as shown in Fig. 2a. The chip layout (Fig. 2b) has a footprint of 9.4 mm × 19.5 mm and a loading pad servicing two equivalent flow paths each comprising a hydraulic flow resistor and a 420-nL-volume capillary pump with 60-µm-diameter pillars arranged in a hexagonal lattice. A pair of electrodes were patterned along each capillary pump and connected to contacts mating with the microSD connector. Electrode pairs had also extensions in the loading pad and at the end of the pump to detect the addition of a pipetted liquid to the loading pad and the complete filling of the pump, respectively. The “chip-olate” process developed in our group^30^ was used to perform all fabrication steps at a wafer-level and singulate sealed and “ready-to-use” chips by simply breaking the fabricated wafer like a chocolate bar (Fig. 2c). In addition, semi-circular structures (“anti-wetting structures”) patterned around the loading pad helped pinning a liquid in the loading pad once pipetted there. Except for the loading pads, chips were sealed by lamination of rectangular strips of dry-film resist (DFR), which made sealing efficient, fast, low-cost, and free of any photolithographic, mechanical patterning, or bonding step (Fig. 2d). Figure 2e shows a microfluidic chip plugged to the peripheral device after the sealing and singulation steps.

### Electrical and thermal characterization of the Arduino-based portable device

**Figure 3:**
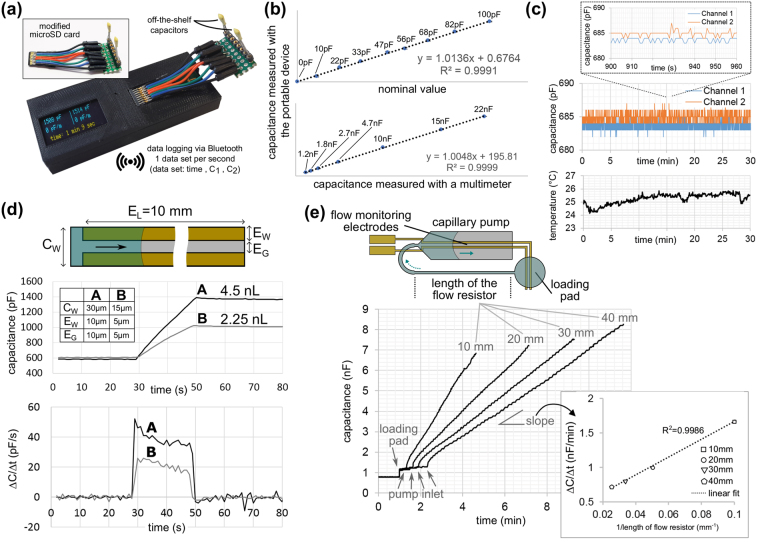
Results of characterization experiments. (**a**) Photograph of the flow monitoring device with a modified microSD memory card allowing electrical connections to off-the-shelf capacitors. (**b**) Graphs showing the correlation of the measured capacitance values to nominal values of smaller capacitors (<100 pF) and to values of larger capacitors (>1 nF) measured using a multimeter. (**c**) Capacitance measurements of a capillary-driven microfluidic chip in dry state, showing zero drift in 30 min and <3 pF peak-to-peak variation, and the temperature of the microfluidic chip measured using an infrared thermopile, showing about 1.5 °C increase during continuous capacitance measurements. (**d**) Measured capacitance and the calculated change in the capacitance during the capillary flow of PBS with 1% BSA and 0.05% Tween 20 for two straight channels having different channel and electrode dimensions. (**e**) Flow rate estimation using capacitance measurements for 4 designs with different flow resistors (50 µm wide and 10, 20, 30, 40 mm long, the flow path is illustrated using the simplified chip layout on top). Inset: Plot showing the slope of the change in the capacitance in the capillary pump with respect to the reciprocal of the length of the flow resistor.

The electrical performance of the portable device was first evaluated using of-the-shelf capacitors and the setup shown in Fig. 3a. We compared values measured by our device to nominal values of the capacitors and to those measured by a commercial multimeter. Plots given in Fig. 3b show that the portable device provides a highly linear (R^2^ > 99%) response for capacitance values that are relevant for monitoring flow in microfluidic chips used in this work (0 pF to 22 nF). Long-term drift, electronic noise, and temperature sensitivity were measured using both a microfluidic chip in dry-state (Fig. 3c) and off-the-shelf capacitors having 4 different values (100 pF, 1, 10, and 47 nF) (see Supplementary Fig. S2a,b). We observed that the drift during a 30 min of recording was detectable only for capacitances larger than 1 nF and it was only 0.5% of the initial value. The maximum peak-to-peak variation was 2 pF for capacitances smaller than 1 nF and less than 0.1% for larger capacitance values (>10 nF). The temperature experienced by the microfluidic chip increased only by 1.5 °C during 30 min of operation and therefore should not influence bioassays performed on chips. If needed, this slight increase in the temperature, which mainly comes from the microcontroller, can be decreased by reducing the measurement frequency or by sending raw data to the smartphone for processing. We also placed the device on a hot plate at 65 °C and inside a fridge at 6 °C to test the effect of ambient temperature on the device performance (see Supplementary Fig. S2c). An elevated temperature slightly increased the noise and drift of measured capacitance values by less than 1% for a 1 nF capacitor. Cooling the device to 6 °C resulted in a more significant change (2.5%) probably due to suboptimal internal calibration of the on-chip temperature sensor of the microcontroller for lower temperatures.

### Characterization of the electrode geometries and the capillary-driven flow

The position, orientation and geometry of the electrodes play an important role for the accuracy of the measurements and the uniformity of the capillary flow. We first characterized the capillary flow of PBS with 1% BSA and 0.05% Tween 20 (a standard buffer used in biochemistry) using image processing (see Supplementary Fig. S3). The results suggested that electrodes placed in the middle of the capillary pump and parallel to the flow path would give a good approximation of the total liquid volume displaced without adversely affecting the capillary flow. Electrodes patterned orthogonal to the flow direction, however, resulted in a non-uniform filling of the pump because the liquid front occasionally got pinned at the electrode edges (see Supplementary Fig. S4). We then tested the effect of the width and the gap of the electrode pair. The measured capacitance scaled linearly with the electrode width because the C_dl_ is proportional to the electrode area (see Supplementary Fig. S5). As a rule of thumb, a wider electrode would give a higher sensitivity and better representation of the liquid position; however, the higher capacitance would take more time to measure, resulting in a potential latency for fast-flowing liquids. We chose 60 µm width and 140 µm gap for the failure detection experiments explained in the next section. It is also possible to replace the external charging resistor (R_c_) with a tunable one (e.g. a digital potentiometer or a transistor as a variable resistor) and control the charging time dynamically (i.e. auto-ranging) depending on the flow conditions and the electrode width.

We then characterized the resolution of our flow monitoring technique using two straight microfluidic channels having 4.5 nL and 2.25 nL volumes, respectively (see Supplementary Fig. S6 for experimental details). Figure 3d shows the measured capacitance when the pipetted buffer solution reaches the measurement channel in about 30 s, advances in the 10-mm-long straight channel for 20 s and then completely stops. The change in the capacitance was 178 pF per nL of displaced liquid. Based on this result and the measured electronic noise of <3 pF, we concluded that the system can resolve liquid displacements as small as 17 pL. For a sampling rate of 1 data/s, this would correspond to a flow rate of 17 pL/s (or ~1 nL/min). For applications that may require a better resolution, using shallower microfluidic channels (<15 µm deep) and a microcontroller with a better ADC performance could theoretically give sub-pL resolution.

**Figure 4:**
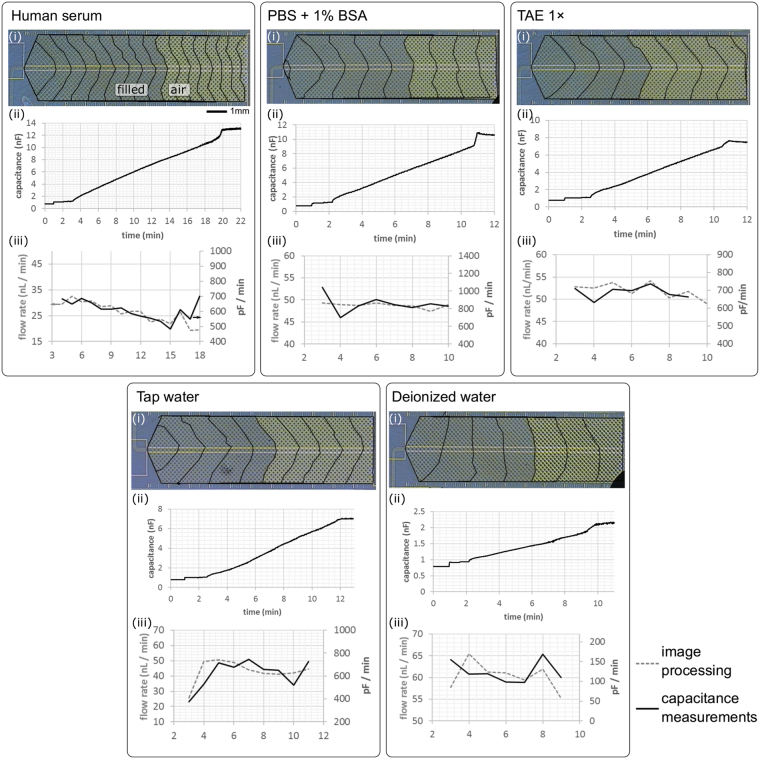
Flow monitoring for various aqueous-based solutions. For each liquid, three experimental results are provided: (i) microscope image of the capillary pump showing the position of the liquid front traced every minute, (ii) the plot showing the capacitance value measured continuously using the portable device, and (iii) the flow rate information calculated using both image processing (dashed line) and capacitance measurements (straight line). For each experiment, measured capacitance increases cumulatively when the chip is inserted, the liquid is pipetted (electrodes in the loading pad), and it advances in the capillary pump. Finally, the capacitance reaches to a plateau after a sharper change in the capacitance owing to additional electrodes placed at the end of the pump.

Experiments for the flow rate estimation were performed using chips with identical capillary pumps and electrodes but having 4 different flow resistors, which are defined by the total length of the channel between the loading pad and the capillary pump. The plots given in Fig. 3e show that the delay introduced by different flow resistors can easily be detected from the points where the capacitance starts increasing at the pump inlet (highlighted with arrows). Also, the change in the capacitance during the liquid flow was inversely proportional to the flow resistance, suggesting that the system would have a linear response to different flow rates of the same liquid. We then tested the compatibility of the technique with different aqueous-based solutions, namely human serum, PBS and TAE buffers, tap water and deionized water. Figure 4 shows microscope snapshots obtained during capillary filling of the pumps and the corresponding capacitance measurements. For each liquid, the flow rates calculated using the electrical measurements were compared to those calculated using image processing. The results from both techniques were in good agreement except for the beginning of the pump where the tapered profile of the pump inlet resulted in a faster linear displacement of liquid at the center. We could observe that human serum flows slower than deionized water, as expected based on the higher viscosity of human serum compared to that of deionized water (see Supplementary Video 1 and Supplementary Fig. S8). In addition, the flow rate was nearly constant (50–60 nL/min) during capillary filling of the pump for all liquids.

### Failure detection and smartphone communication for enhanced interactivity with the user

**Figure 5:**
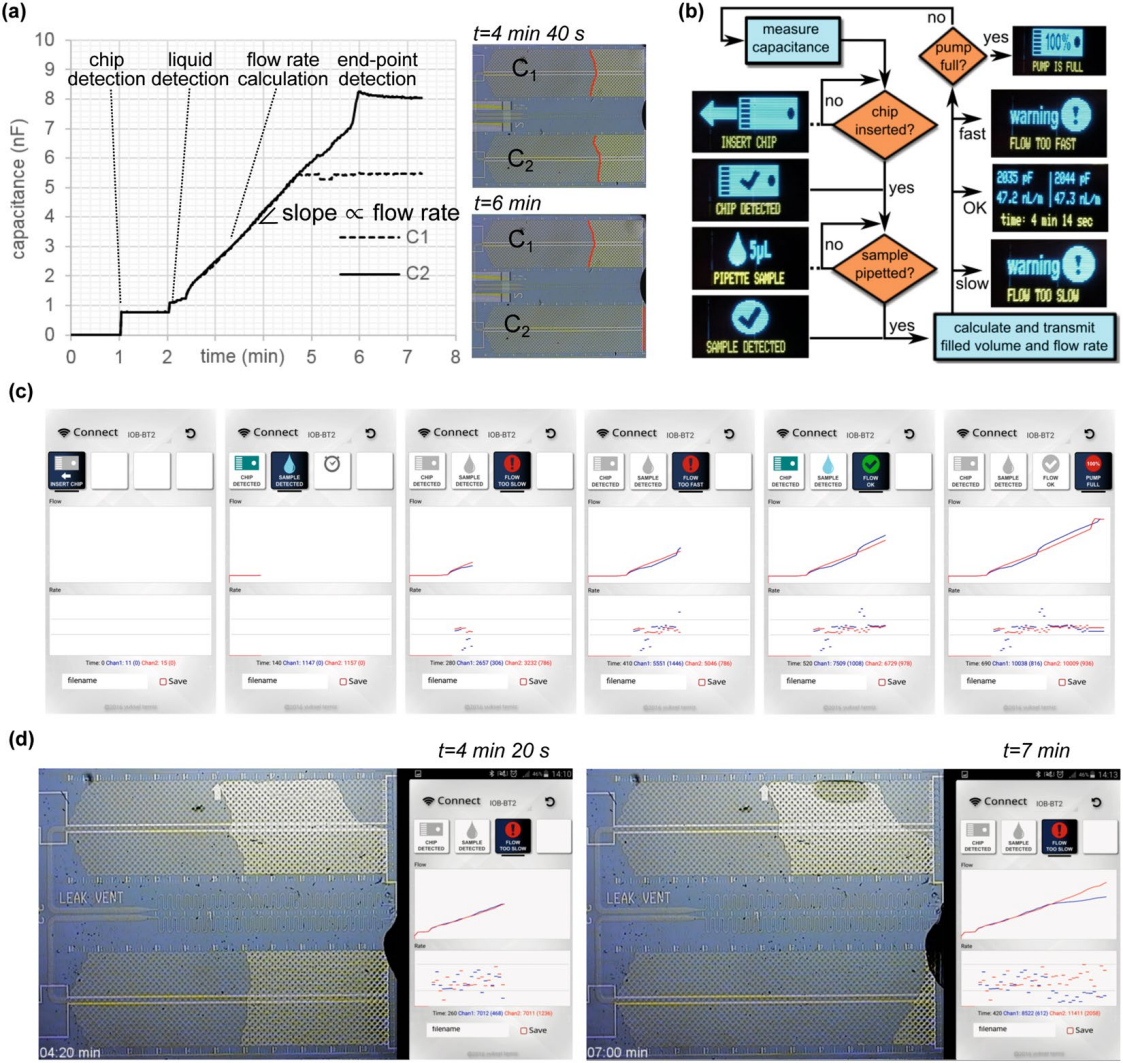
Algorithm and smartphone interface for real-time flow monitoring and failure detection. (**a**) Microscope images and capacitance values recorded from two channels during the flow of PBS with 1% BSA and 0.05% Tween 20 (the flow of the top channel was intentionally stopped by blocking its air vent). (**b**) Simplified sketch of the algorithm used to monitor chip insertion, sample pipetting, and various anomalies in the flow. (**c**) Snapshots from the smartphone app showing the flow conditions and warnings in real-time for a simulated data sent by the portable device. (**d**) Microscope images during the capillary filling of a chip having defects in the upper channel and snapshots from the smartphone app that shows the flow information in real-time and warns the user promptly when a failure occurs.

Having verified the microfluidic functionality and the device operation, we tested the algorithm and the smartphone app developed for real-time detection of various anomalies in the flow and for guiding the user. First, we defined the distinct changes in the capacitance related to the insertion of the chip, liquid flow in the pump, and presence of the liquid in the loading pad and the end of the pump. We also simulated a failure (e.g. clogging, leakage to the vent) by blocking the air vent of a channel. Figure 5a shows the capacitance values from two microfluidic channels, giving distinguishable changes in the capacitance for conditions mentioned above (e.g. 200 pF for liquid detection). Figure 5b then shows the simplified illustration of the algorithm defining different “states” based on the measured capacitance and pre-defined threshold values for each condition (see the Methods section and Supplementary Fig. S9 for details). The OLED display on the device and the smartphone app then display the icons/warnings based on the state information and the measured data. Figure 5c shows snapshots from the smartphone app where the data recorded from a microfluidic chip, which was modified to simulate typical failure scenarios (flow slower or faster than ideal). This experiment verified that the device recognizes various failures in real-time and the phone warns the user via changing icons and vibration. We then tested an actual failure where one of the channels had a leakage to the capillary pump then eventually to the air vent due to a defect in the laminated cover film. Snapshots from this experiment are shown in Fig. 5d and Supplementary Video 2.

## Discussion

We demonstrated a chip design and a portable peripheral device for real-time, high-resolution, and multiplexed monitoring of liquid flow in microfluidics using capacitance measurements. Monitoring uses integrated electrodes that are directly in contact with the liquid, i.e. no passivation or surface modification is required. The device can monitor the flow from two independent microfluidic flow paths, but the number of channels can easily be increased by connecting more contact pads to the microcontroller or adding an analog multiplexer. Because the capacitance measurement technique uses a generic Arduino microcontroller and requires only two external resistors, all parameters (e.g. resolution, measurement speed, applied voltage) can be changed from the software (or from the smartphone) depending on the chip architecture and the application. In addition to flow rate measurements, we implemented a simple algorithm that compares the measured capacitance values to preset thresholds defining different flow conditions to guide the user (e.g. informing when to read the data, warning if there is a failure, etc). Such an interaction with the user is particularly important for rapid diagnostic tests because it could potentially minimize user-related errors and minimize subjective interpretation of assay results. Calibration of the assay result based on the measured flow rate could reduce large error bars typically observed in such tests. Because the standalone peripheral is as small as a lateral flow assay (see Supplementary Fig. S1d), it can be used with existing portable readers or smartphone attachments designed to readout those assays. Multiple units of such compact and smart flow monitoring peripherals would also allow running many tests in parallel without occupying the reader during the whole assay time.

Although the system works well with aqueous solutions commonly used in biological and chemical assays, one limitation of the current implementation is that the user needs to calibrate the system for a particular liquid (i.e. the device cannot estimate the flow rate of a liquid with unknown electrical properties). In the future, a database can be implemented, so the user would just select the chip version, the application, and type of the liquid (e.g. urine, human serum), or read a QR code so the threshold values for the algorithm are automatically set. More preferably, a self-calibration mechanism can be implemented where a small side channel connected to the loading pad quickly measures the electrical characteristics of the liquid and applies the values for the flow rate calculations in the main channel.

Many microfluidic devices already use integrated electrodes for electrical detection or liquid/particle manipulation, such as electroosmotic flow, electrowetting, and dielectrophoresis, but they still rely on optical feedback for the flow rate estimation or failure detection. Our technique can be readily implemented in those applications without changing the chip fabrication process or the assay readout. As an example, we recently published a new technique to control the capillary flow using electrically-actuated valves and integrated electrodes^31^. The combination of flow control and monitoring using such electrodes would give the possibility to develop versatile microfluidic chips where the flow profile is defined via a smartphone and actual flow rate is measured in real-time. We demonstrated the concept in Si-based capillary-driven microfluidics fabricated using standard cleanroom processes, but electrodes can be integrated nowadays as well in other substrates that are commonly used in LoC devices, such as low-cost plastics^32^, printed circuit boards^33^, 3D-printed^34^ and inkjet-printed^35^ microchannels, and even paper-based devices^36^. There is already a substantial effort to integrate elements for flow manipulation or electrical detection in low-cost paper microfluidics^37^. Paper-based assays might as well benefit from precise flow monitoring for device characterization and assay validation. In addition to passive microfluidic systems, the principle can also be applied to microfluidic applications using external energy for liquid pumping. For example, priming of an electroosmotic pump, bubble-free filling of microfluidic channels in pressure-driven systems, or liquid handling steps in Lab-on-a-Disc systems could be monitored without using an optical setup. Because capacitance-based detection is fast (less than 100 ms per measurement) and sensitive (3 pF resolution), a similar peripheral device could also be employed to monitor segmented flows, or flow of cells using electrode pairs patterned perpendicular to the flow direction.

Variants of this generic and easy-to-implement flow monitoring concept can be readily applied to many microfluidics-based applications to realize next-generation POCD devices that are not only portable and reliable but also smart, connected and interactive.

## Methods

### Fabrication of capillary-driven microfluidic chips

Chips were fabricated on standard 4-inch Si wafers (Si-Mat, Germany) with 200 nm thermally-grown SiO_2_ using metal lift-off process for electrodes and SU-8 patterning for microfluidic structures (Fig. 2c). First, a positive-tone photoresist (AZ®4562, MicroChemicals GmbH) was patterned for the electrodes using standard UV photolithography. Prior to metal deposition, the SiO_2_ layer was etched to 50–55 nm depth in buffered hydrofluoric acid (BHF) using the photoresist layer as the mask. This partial wet etching step facilitates the subsequent metal lift-off process and enables electrodes embedded in the SiO_2_ layer; thus, the surface topography is minimized for a more uniform capillary flow. 5-nm-thick Ti (adhesion layer) and 50-nm-thick Pd (electrode layer) were deposited using e-beam evaporation. The lift-off process was then performed in acetone followed by rinsing in isopropyl alcohol (IPA). Following a 2-min O_2_ plasma treatment (600 W, 300 sccm), a 15-µm-thick SU-8 (SU-8 3010, MicroChem Corp.) was patterned for the microfluidic structures using the recipe provided by the supplier. The wafer surface was protected using AZ®4562 photoresist and the wafer was partially diced to half of its thickness using a semi-automated dicing tool. Following wafer-level photoresist cleaning in acetone and IPA, a 50-µm-thick dry-film resist (DFR, DF-1050 from Engineered Materials Systems Inc., USA) was cut to rectangular strips using a scissor, aligned to anti-wetting structures in SU-8, and laminated at 45 °C. Finally, “ready-to-use” chips were singulated by manual breaking using the “chip-olate” process^30^.

### Preparation of liquid samples

Deionized water was obtained using a Millipore synergy UV system (Merck Millipore Ltd., USA). Phosphate-buffered saline (PBS) was prepared by dissolving tablets (P4417, Sigma-Aldrich) and filtering the solution using a 0.22 µm filter (Merck Millipore Ltd.). Bovine serum albumin (BSA, Sigma-Aldrich) was dissolved at a concentration of 1% w/v in PBS. TAE (Tris-acetate-EDTA) 1× was obtained by diluting the 10× stock solution (T9650, Sigma-Aldrich). Human serum (H4522) was purchased from Sigma-Aldrich. Although it was not required for the capillary filling, 0.05% (v/v) Tween® 20 surfactant was added to all solutions to obtain a more uniform liquid filling of the capillary pump and to mimic the conditions used in typical biochemical assays. All solutions were prepared and used at room temperature.

### Implementation of Arduino-based peripheral devices

Three portable devices using the same capacitance measurement algorithm but having different dimensions and battery capacities were designed and implemented (see Supplementary Fig. S1). For the first two devices (Device 1 and 2), we used a commercial Arduino board (Pro Micro 3.3 V/8 MHz from SparkFun Electronics) and a standard OLED display with a SSD1306 driver chip. For the more compact Device 3, we designed a custom printed circuit board without the display option. Bluetooth modules (HC-05 or RN-42) were connected to the Arduino boards via the serial universal asynchronous receiver/transmitter (UART) bus. OLED displays with 128 × 64 (Device 1) and 128 × 32 (Device 2) pixels were connected to the microcontroller via the I^2^C bus. 3.7 V, 400 mAh (Device 1) and 110 mAh (Device 2 and 3) rechargeable LiPo batteries were used to supply power to the whole system. A charge management controller chip (MCP73831 from Microchip) was used to charge the batteries via the USB interface. Electrical connection to the microfluidic chip was achieved by removing the metal casing of an 8-contact microSD memory card connector (MOLEX 502774-0891). For capacitance measurements, only two external resistors per channel were needed: 10 MΩ for charging and 1 KΩ for discharging the unknown capacitance. Components were assembled on a 2-layer printed circuit board and fit into an ABS housing designed using FreeCAD software and printed using Dimension Elite 3D printer.

### Arduino code for capacitance measurements and failure detection

The code running on the Arduino microcontroller was written using the standard Arduino IDE (v1.6.13) software. The code required installation of three additional libraries, “Wire.h”, “Adafruit_GFX.h” and “Adafruit_SSD1306.h”, to display text and graphics on the OLED display. The unknown capacitance was calculated by measuring the time required to charge it from 0 V to 1 V by continuously reading the analog voltage on the capacitor using a “while” loop, which breaks when the voltage reaches to 1 V. The capacitor voltage was then set back to 0 V to make it ready for the next measurement (see Supplementary Fig. S1b for microcontroller instructions). To improve the resolution of the measurements, default settings of the 10-bit ADC of the microcontroller were modified. ADC high speed mode was activated by setting the ADCHSM register to logic “1” and the reference value of the ADC was set to “INTERNAL”, 2.56 V, instead of the default supply voltage of 3.3 V. Ten consecutive capacitance measurements were averaged for each data point and change in the capacitance (i.e. rate) was calculated every 10 s. The calculated values from two microfluidic channels were compared to preset thresholds defining 7 different states (see Supplementary Fig. S9 for the full algorithm and definitions of the states). Finally, the data set comprising the state, the time, the capacitance and the rate values were sent to the display and the Bluetooth module every second using the “Serial.print()” function.

### Android app for flow monitoring and interactivity with the user

An Android app was developed to display the flow information and warn the user when a failure is detected (see Supplementary Fig. S10). Here, we had two options depending on where the failure detection algorithm is executed: (i) the peripheral device executes the algorithm and sends the final information to the smartphone or (ii) the peripheral device sends the raw values (measured charging time) and smartphone executes the algorithm. We preferred the first option because the peripheral device could be used in the stand-alone mode without requiring a continuous Bluetooth communication with the phone. The smartphone app was written in Java script using the DroidScript (http://droidscript.org) platform. The incoming data string from the Bluetooth channel was parsed into separate variables. The “state” value was used to display various icons as shown in Fig. 5c. The values for the time, the capacitance, and the rate were used to draw lines in the plot area representing the filled volume and the flow rate. We also added buttons to save the raw data to a text file, to reset timers and clear plots, and to activate the continuous sampling mode, which ignores the failure detection algorithm and displays the incoming data regardless of the state value (this mode was used to record raw data during the characterization experiments given in Figs 3 and 4).

### Electrical and thermal characterization

Off-the-shelf capacitors with known values were used to measure the resolution and the temperature sensitivity of the capacitance measurement technique (Fig. 3a–c and Supplementary Fig. S2). A custom electrical connector was implemented by cutting the connections of a microSD memory card and soldering them to a pin header. The parasitic capacitance coming from the board connections and the connector, which was around 40 pF, was subtracted from the measured value. Measured capacitance values were compared to those measured using a multimeter (PeakTech 2025), which could not measure capacitances less than 1 nF; thus, nominal values were used for smaller capacitances. For drift and noise measurements, capacitances from two channels were recorded for 30 min at 1 data/s rate. All electrical characterization experiments were conducted at 25 °C. For the thermal characterization experiments, the peripheral device was placed on a hot plate (25–60 °C) and then inside a fridge at 6 °C without interrupting the data recording. The change in the temperature of the plugged microfluidic chip due to the activity of the microcontroller was measured using an infrared temperature sensor (TMP007 from Texas Instruments) connected to another Arduino board with a Bluetooth module. Capacitance values and temperature measurements were recorded simultaneously by opening two Bluetooth channels in RealTerm serial terminal software running on a laptop with Windows operating system.

### Flow rate calculation based on image processing

Snapshot images of the microfluidic chip were captured every minute during the flow monitoring experiments using a digital camera attached to a Leica MZ16 stereomicroscope, having an overall field of view of 9 × 12 mm^2^. The meniscus of the liquid advancing in the capillary pump was traced manually using the ImageJ software. Here, the optical contrast between the filled and unfilled regions were not strong enough for an automatic edge detection in good precision. Knowing the effective area of each capillary pump from the layout (measured 27.8 mm^2^ using L-Edit layout editor) and the actual depth of fabricated channels (measured 15 µm using Veeco Dektak 6 M profilometer), the total liquid capacity of the pump was calculated as 420 nL. The filled volume and the flow rate were then calculated by scaling the area calculated by ImageJ to the actual area, thus total volume.

## Electronic supplementary material

Supplementary Information

Flow monitoring experiments using human serum

Smartphone interface for real-time flow monitoring and failure detection

## References

[1] Yang K, Peretz-Soroka H, Liu Y, Lin F (2016). Novel developments in mobile sensing based on the integration of microfluidic devices and smartphones. Lab Chip.

[2] Xu X (2015). Advances in smartphone-based point-of-care diagnostics. Proc. IEEE.

[3] McCracken KE, Yoon J-Y (2016). Recent approaches for optical smartphone sensing in resource-limited settings: a brief review. Anal. Methods.

[4] Perkel, J. M. Pocket laboratories. *Nat*. 2017 5457652 (2017). 10.1038/545119a 28470200

[5] Qin C (2016). The assessment of the readiness of molecular biomarker-based mobile health technologies for healthcare applications. Sci. Rep..

[6] Lafleur JP, Jönsson A, Senkbeil S, Kutter JP (2016). Recent advances in lab-on-a-chip for biosensing applications. Biosens. Bioelectron..

[7] Temiz, Y., Lovchik, R. D., Kaigala, G. V. & Delamarche, E. Lab-on-a-chip devices: How to close and plug the lab? *Microelectron*. *Eng*. **132** (2015).

[8] Posthuma-Trumpie GA, Korf J, van Amerongen A (2009). Lateral flow (immuno)assay: its strengths, weaknesses, opportunities and threats. A literature survey. Anal. Bioanal. Chem..

[9] Gervais L, de Rooij N, Delamarche E (2011). Microfluidic chips for point-of-care immunodiagnostics. Adv. Mater..

[10] Boyd-Moss M, Baratchi S, Di Venere M, Khoshmanesh K (2016). Self-contained microfluidic systems: a review. Lab Chip.

[11] WHO | Malaria rapid diagnostic test performance: results of WHO product testing of malaria RDTs: round 6 (2014–2015). WHO (2016).

[12] Miller E, Sikes HD (2015). Addressing barriers to the development and adoption of rapid diagnostic tests in global health. Nanobiomedicine.

[13] St John A, Price CP (2014). Existing and emerging technologies for point-of-care testing. Clin. Biochem. Rev..

[14] Nguyen N (1997). Micromachined flow sensors—a review. Flow Meas. Instrum..

[15] Zarifi MH, Sadabadi H, Hejazi SH, Daneshmand M, Sanati-Nezhad A (2018). Noncontact and Nonintrusive Microwave-Microfluidic Flow Sensor for Energy and Biomedical Engineering. Sci. Rep..

[16] Alfadhel A (2014). A magnetic nanocomposite for biomimetic flow sensing. Lab Chip.

[17] Noeth N, Keller S, Boisen A (2013). Integrated cantilever-based flow sensors with tunable sensitivity for in-line monitoring of flow fluctuations in microfluidic systems. Sensors.

[18] Zhang L (2015). Highly sensitive microfluidic flow sensor based on aligned piezoelectric poly(vinylidene fluoride-trifluoroethylene) nanofibers. Appl. Phys. Lett..

[19] Collins J (2004). Microfluidic flow transducer based on the measurement of electrical admittance. Lab Chip.

[20] Wu J (2005). Micro flow sensor based on two closely spaced amperometric sensors. Lab Chip.

[21] Arjmandi N, Liu C, Van Roy W, Lagae L, Borghs G (2012). Method for flow measurement in microfluidic channels based on electrical impedance spectroscopy. Microfluid. Nanofluidics.

[22] Bathany C, Han J-R, Abi-Samra K, Takayama S, Cho Y-K (2015). An electrochemical-sensor system for real-time flow measurements in porous materials. Biosens. Bioelectron..

[23] Luo X, Davis JJ (2013). Electrical biosensors and the label free detection of protein disease biomarkers. Chem. Soc. Rev..

[24] Wyatt Shields IV C, Reyes CD, López GP (2015). Microfluidic cell sorting: a review of the advances in the separation of cells from debulking to rare cell isolation. Lab Chip.

[25] Feng S, Tseng D, Di Carlo D, Garner OB, Ozcan A (2016). High-throughput and automated diagnosis of antimicrobial resistance using a cost-effective cellphone-based micro-plate reader. Sci. Rep..

[26] Guo J (2016). Uric acid monitoring with a smartphone as the electrochemical analyzer. Anal. Chem..

[27] Sun A, Wambach T, Venkatesh AG, Hall DA (2014). A low-cost smartphone-based electrochemical biosensor for point-of-care diagnostics. Proc. IEEE Biomed. Circuits Syst. Conf..

[28] McCracken KE, Angus SV, Reynolds KA, Yoon J-Y (2016). Multimodal imaging and lighting bias correction for improved μPAD-based water quality monitoring via smartphones. Sci. Rep..

[29] Franks W, Schenker I, Schmutz P, Hierlemann A (2005). Impedance characterization and modeling of electrodes for biomedical applications. IEEE Trans. Biomed. Eng..

[30] Temiz, Y. & Delamarche, E. ‘Chip-olate’ and dry-film resists for efficient fabrication, singulation and sealing of microfluidic chips. *J*. *Micromechanics Microengineering* **24** (2014).

[31] Arango, Y., Temiz, Y., Gökçe, O. & Delamarche, E. Electrogates for stop-and-go control of liquid flow in microfluidircs. *Appl*. *Phys*. *Lett*. **112** (2018).

[32] Zhao B (2017). A controllable and integrated pump-enabled microfluidic chip and its application in droplets generating. Sci. Rep..

[33] Moschou D, Tserepi A (2017). The lab-on-PCB approach: tackling the μTAS commercial upscaling bottleneck. Lab Chip.

[34] Duarte LC, Chagas CLS, Ribeiro LEB, Coltro WKT (2017). 3D printing of microfluidic devices with embedded sensing electrodes for generating and measuring the size of microdroplets based on contactless conductivity detection. Sensors Actuators B Chem..

[35] Su W, Cook BS, Fang Y, Tentzeris MM (2016). Fully inkjet-printed microfluidics: a solution to low-cost rapid three-dimensional microfluidics fabrication with numerous electrical and sensing applications. Sci. Rep..

[36] Ko H (2014). Active digital microfluidic paper chips with inkjet-printed patterned electrodes. Adv. Mater..

[37] Fu E, Downs C (2017). Progress in the development and integration of fluid flow control tools in paper microfluidics. Lab Chip.

