# Supplementary material for: Sub-nanoliter, real-time flow monitoring in microfluidic chips using a portable device and smartphone
Source: Sci Rep. 2018 Jul 13;8:10603. doi: 10.1038/s41598-018-28983-w (PMC6045673; doi:10.1038/s41598-018-28983-w)
Supplement: Supplementary file 1 — Supplementary Information [file 41598_2018_28983_MOESM1_ESM.pdf]

Sub-nanoliter, real-time flow monitoring in microfluidic chips using a portable device and smartphone

- Supplementary Information –

Yuksel Temiz* and Emmanuel Delamarche

IBM Research – Zurich, Säumerstrasse 4, CH-8803 Rüschlikon, Switzerland

*yte@zurich.ibm.com

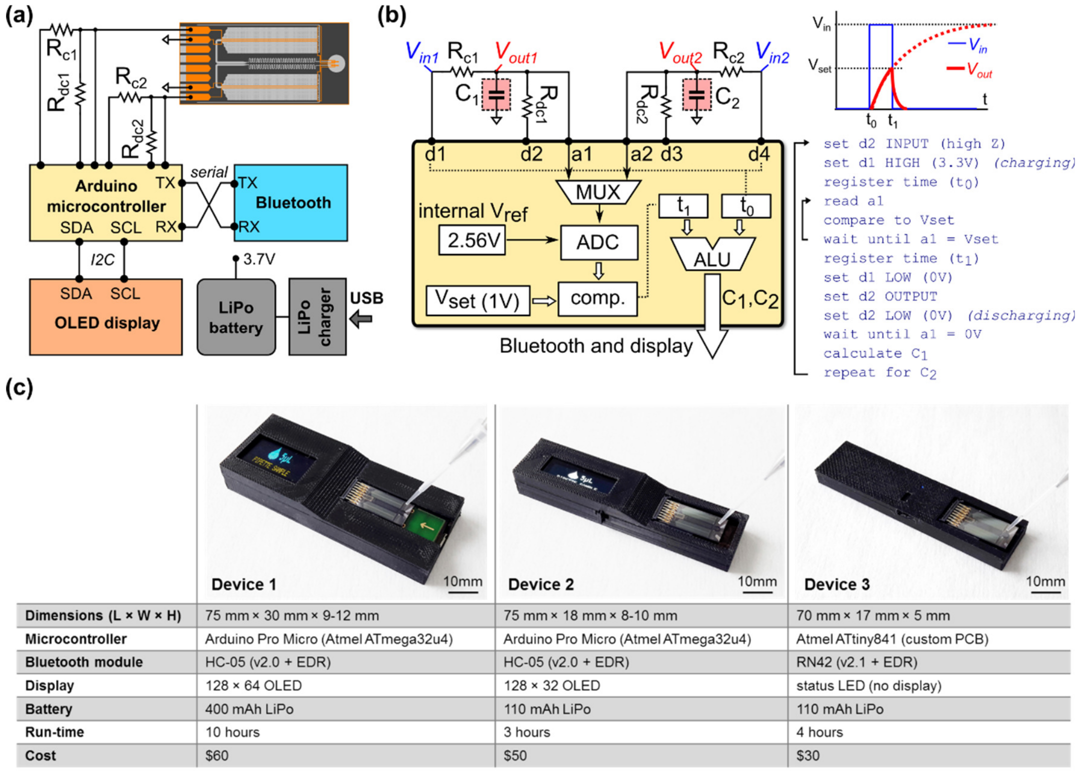

	Device 1	Device 2	Device 3
**Dimensions (L × W × H)**	75 mm × 30 mm × 9-12 mm	75 mm × 18 mm × 8-10 mm	70 mm × 17 mm × 5 mm
**Microcontroller**	Arduino Pro Micro (Atmel ATmega32u4)	Arduino Pro Micro (Atmel ATmega32u4)	Atmel ATtiny841 (custom PCB)
**Bluetooth module**	HC-05 (v2.0 + EDR)	HC-05 (v2.0 + EDR)	RN42 (v2.1 + EDR)
**Display**	128 × 64 OLED	128 × 32 OLED	status LED (no display)
**Battery**	400 mAh LiPo	110 mAh LiPo	110 mAh LiPo
**Run-time**	10 hours	3 hours	4 hours
**Cost**	$60	$50	$30

(d)

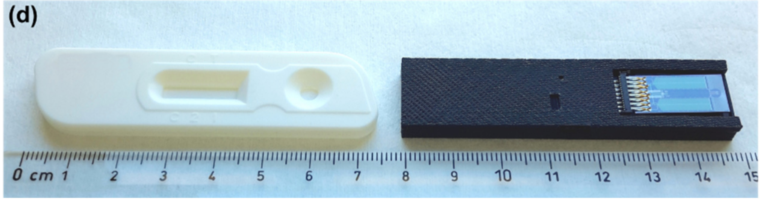

(e)

Device 1	USD
Arduino Pro Micro	20
HC-05 Bluetooth	10
OLED display	8
Li-Po battery	10
Electronic components	2
PCB and 3D printing	10
**Total**	**60**

Supplementary Figure S1. Design and implementation of portable devices for flow monitoring. (a) Simplified circuit diagram of the portable device designed for capacitance measurements from two independent microfluidic channels. The Arduino microcontroller board is connected to a Bluetooth module and to an OLED display via a serial bus and an I2C bus, respectively. A 3.7 V rechargeable lithium polymer (LiPo) battery is connected to the supply input of the Arduino board. A voltage regulator available on the Arduino board supplies 3.3 V to the Bluetooth module and the OLED display. **(b)** Basic operation principle and instructions of the microcontroller. A logic HIGH (3.3 V) is applied from a digital pin (d1) to the unknown capacitor through a charging resistor (R_{c1} = 10 MΩ) and the capacitor voltage is continuously monitored using an analog pin (a1) while an internal timer keeps track of the charging time. When the voltage reaches the preset threshold (V_{set}, e.g. a low voltage of only 1 V to avoid undesired electrochemical effects), the applied voltage is set to logic LOW (0 V) and the charging time is registered. The capacitor is then quickly discharged via the discharging resistor (R_{dc1} = 1 KΩ) to make it ready for the next measurement. The unknown capacitance is calculated from the measured charging time and the known value of the charging resistor. The same capacitance measurement algorithm is applied to the other channel and the data is displayed and/or sent to a mobile device via Bluetooth. More channels can easily be added if sufficient number of input/output pins is available. **(c)** Photographs and main specifications of implemented portable devices. **(d)** Photograph of Device 3 with a microfluidic chip next to a standard lateral flow assay for size comparison. **(e)** An exemplary price list of components used for the assembly of Device 1.

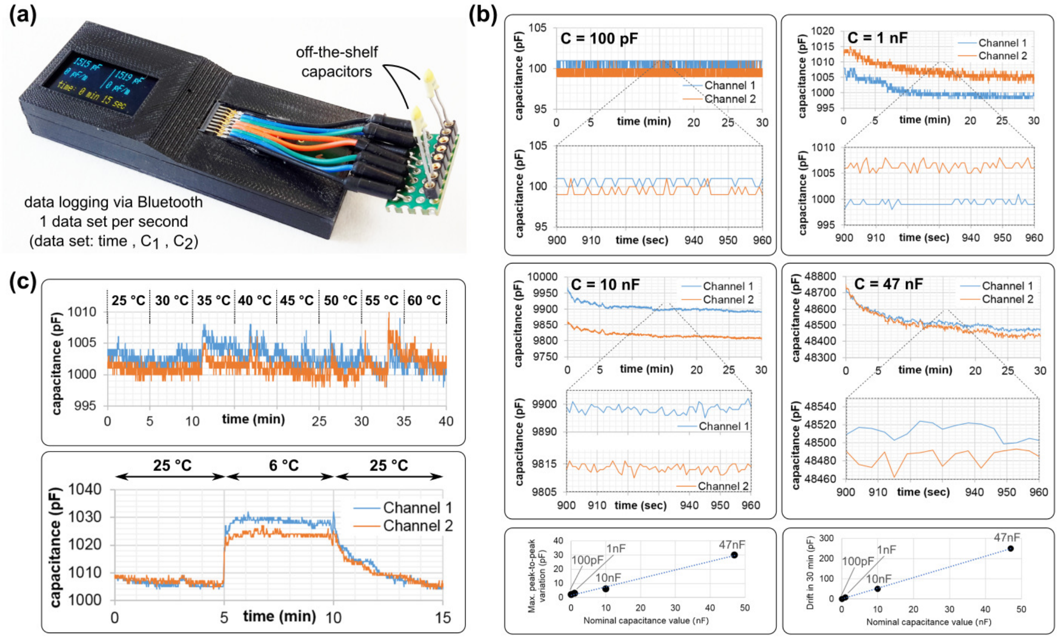

Supplementary Figure S2. Electrical and thermal characterization. (a) Photograph of the flow monitoring device (Device 1) with a modified microSD memory card that allows connections to off-the-shelf capacitors. **(b)** Plots showing the long-term drift (30 min) and peak-to-peak variation (1-min-long subset) for 4 capacitor values. We observed a small drift (less than 0.5%) for capacitors larger than 1 nF. The maximum peak-to-peak variation was 2 pF for smaller values (< 1 nF) and less than 0.1% for large values (> 10 nF). The fact that the noise and the drift scale with the capacitance (plots at the bottom) suggests that the source of the noise might be the microcontroller timer used to measure the charging time. **(c)** Capacitance measurements when the device was placed on a hot plate 25-60 °C (top) and inside a fridge at 6 °C (bottom).

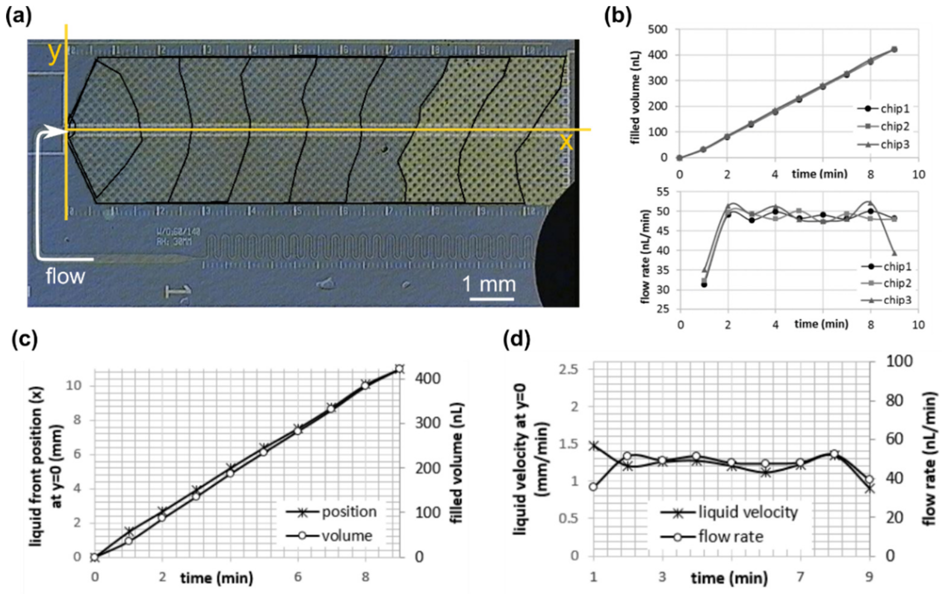

Supplementary Figure S3. Flow characterization using image processing. (a) Microscope image of a microfluidic chip during the capillary flow of PBS with 1% BSA and 0.05% Tween 20. The liquid front advancing in the capillary pump is traced manually using microscope images taken every minute and the ImageJ software. The capillary pump has an area of 27.8 mm^2 and channel depth of 15 µm, which correspond to a total volume of about 420 nL. **(b)** Plots showing the filled volume and the flow rate for 3 chips, where chip-to-chip variation in the flow rate is less than 5% on average. **(c)** Plot showing the correlation between the filled volume and the linear displacement of the liquid front at the center of the pump (y = 0), and **(d)** the corresponding linear velocity and the flow rate. This graph suggests that monitoring flow using electrodes in the middle of the pump gives a good approximation of the total volume displaced, except the entrance of the pump because of its tapered profile. This mismatch was taken into consideration during the implementation of failure detection algorithm.

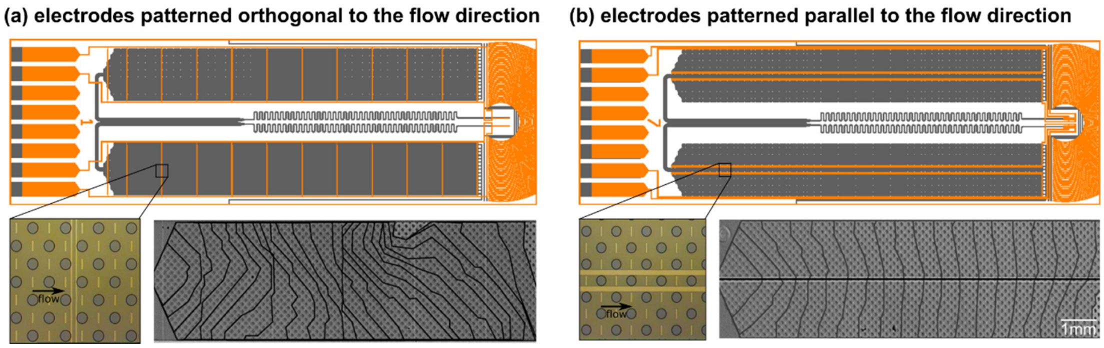

Supplementary Figure S4. Effect of the electrode orientation on the capillary flow. Chip layouts and microscope images showing the advancing of liquid front traced every minute for electrode pairs patterned **(a)** orthogonal and **(b)** parallel to the flow direction. When electrodes are orthogonal to the flow direction, the liquid front may get pinned at the edge of the electrode, resulting in a non-uniform filling of the pump.

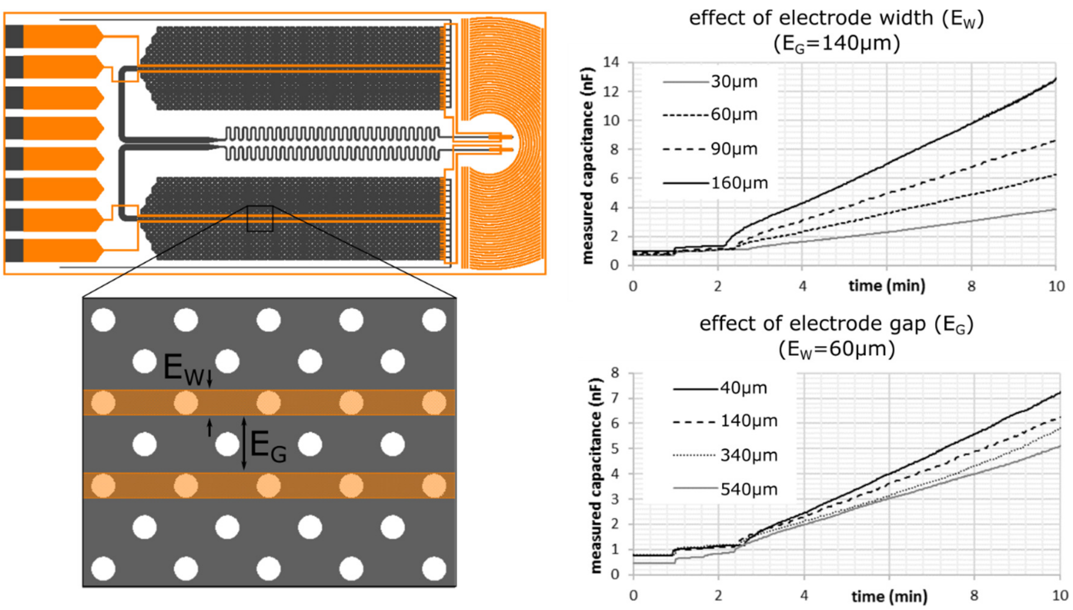

Supplementary Figure S5. Effect of the electrode geometry. Measured capacitance values for different electrode widths and gaps. The capacitance value increases linearly with the electrode width because the double layer capacitance is proportional to the electrode area. The effect of the gap between the electrode pair is less prominent but the measured effective capacitance value increases with the decreasing gap probably due to the contribution of the fringing capacitance, the resistance of the solution, and the charge transfer resistance.

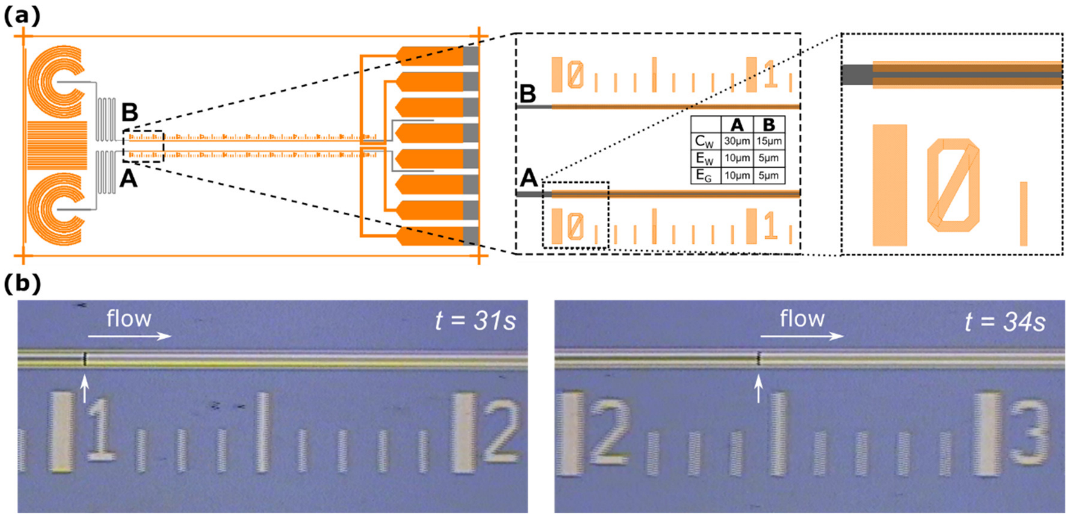

Supplementary Figure S6. Flow monitoring experiments using 10-mm-long straight channels. (a) Layout of the chip design with two independent flow paths. Electrodes are patterned along the flow path with dimensions shown in the table (inset), where C_W is the channel width, E_W is the electrode width, and E_G is the electrode gap. **(b)** Microscope images from design A captured during the capillary filling of PBS with 1% BSA and 0.05% Tween 20. The white arrow points to the liquid front. The ruler is in millimeters.

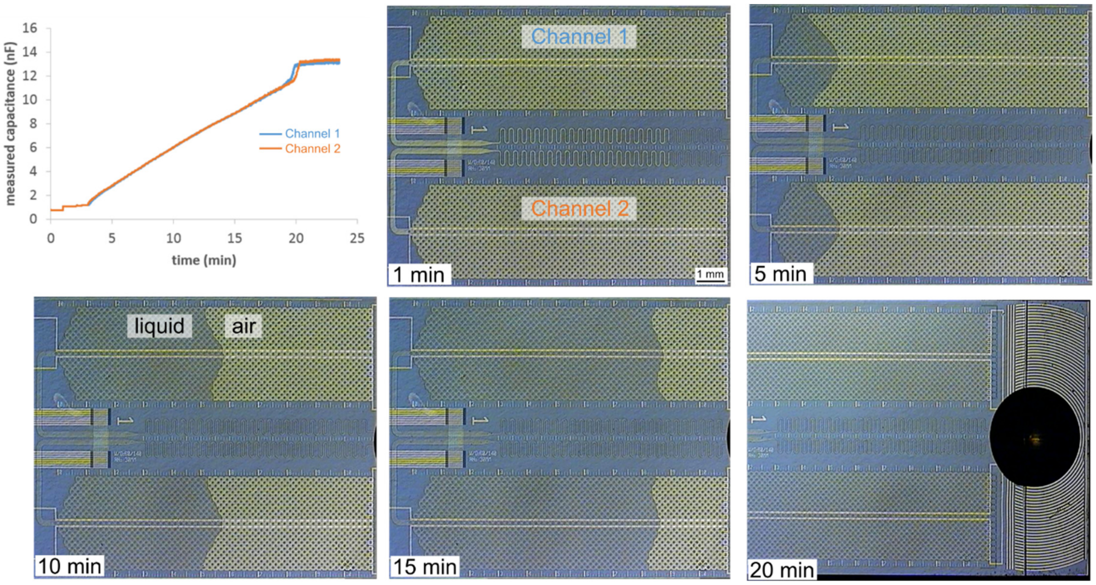

Supplementary Figure S7. Flow monitoring experiments using human serum. Microscope images of the microfluidic chip during capillary filling by human serum having 0.05% Tween 20 and corresponding plot showing the capacitance values measured from two independent flow paths. The last snapshot (bottom right corner) also shows that the sample pipetted onto the loading pad of the chip remained pinned for the entire duration of the experiments due to the presence of semicircular anti-wetting structures.

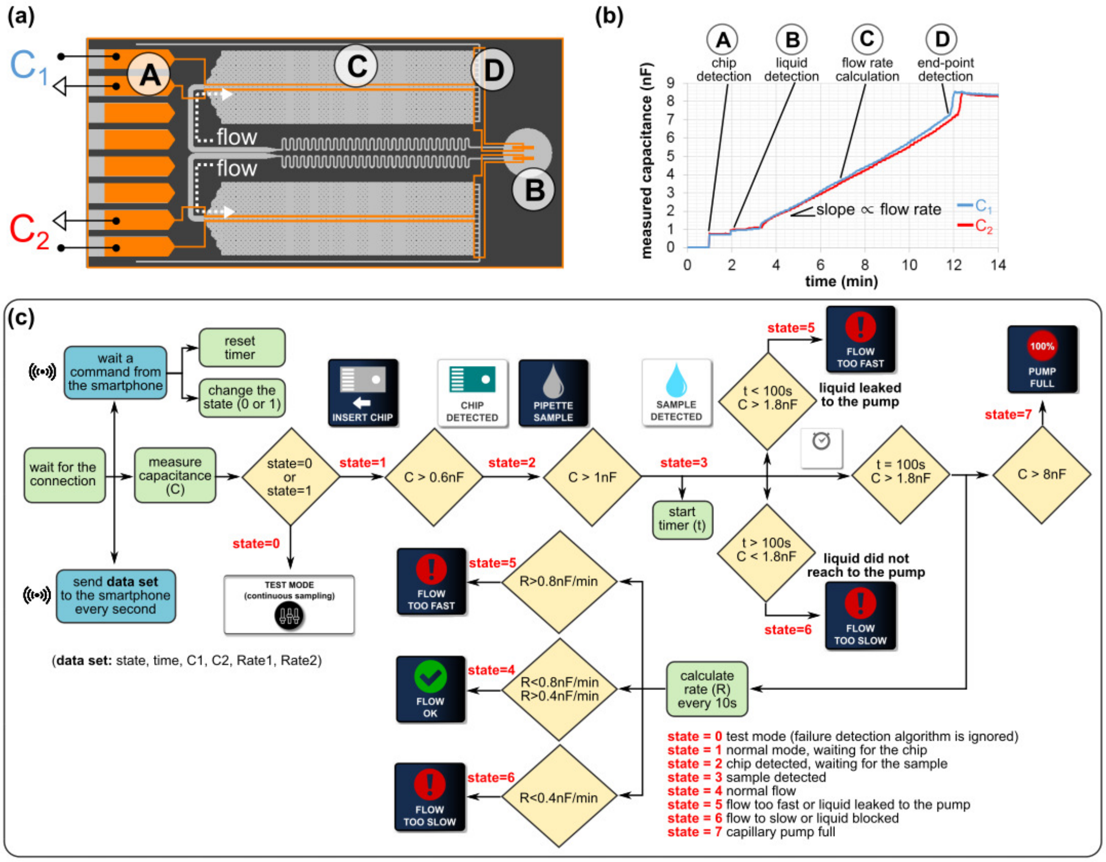

Supplementary Figure S8. Algorithm for continuous failure detection and enhanced interactivity with the user. (a) The chip layout and **(b)** the measured capacitance values showing different check points used in the algorithm. The chip is detected using the dry state capacitance (A), the sample is detected using the electrodes patterned in the loading pad (B), the flow rate is calculated using the electrodes patterned along the capillary pumps (C), and the end-point (full pump) is detected using additional electrodes located at the end of the pump (D). Each microfluidic flow path is monitored using one pair of electrodes (i.e. one capacitance value), therefore, the change in the capacitance is cumulative. **(c)** Flowchart representing the algorithm running on the Arduino microcontroller. There are 7 states defined for different conditions. The algorithm starts the capacitance measurements when the Bluetooth communication is established. The user can reset the timer and the state, and switch between two main states (state = 0: test mode (failure detection algorithm is ignored), state = 1: normal mode (failure detection algorithm is executed)). In the normal mode, the measured capacitance, change in the capacitance (rate measured every 10 s) and the time information are compared to predefined thresholds, then the state is changed accordingly. The information is sent to the smartphone via Bluetooth and/or displayed on the OLED display with 1 s refresh rate.

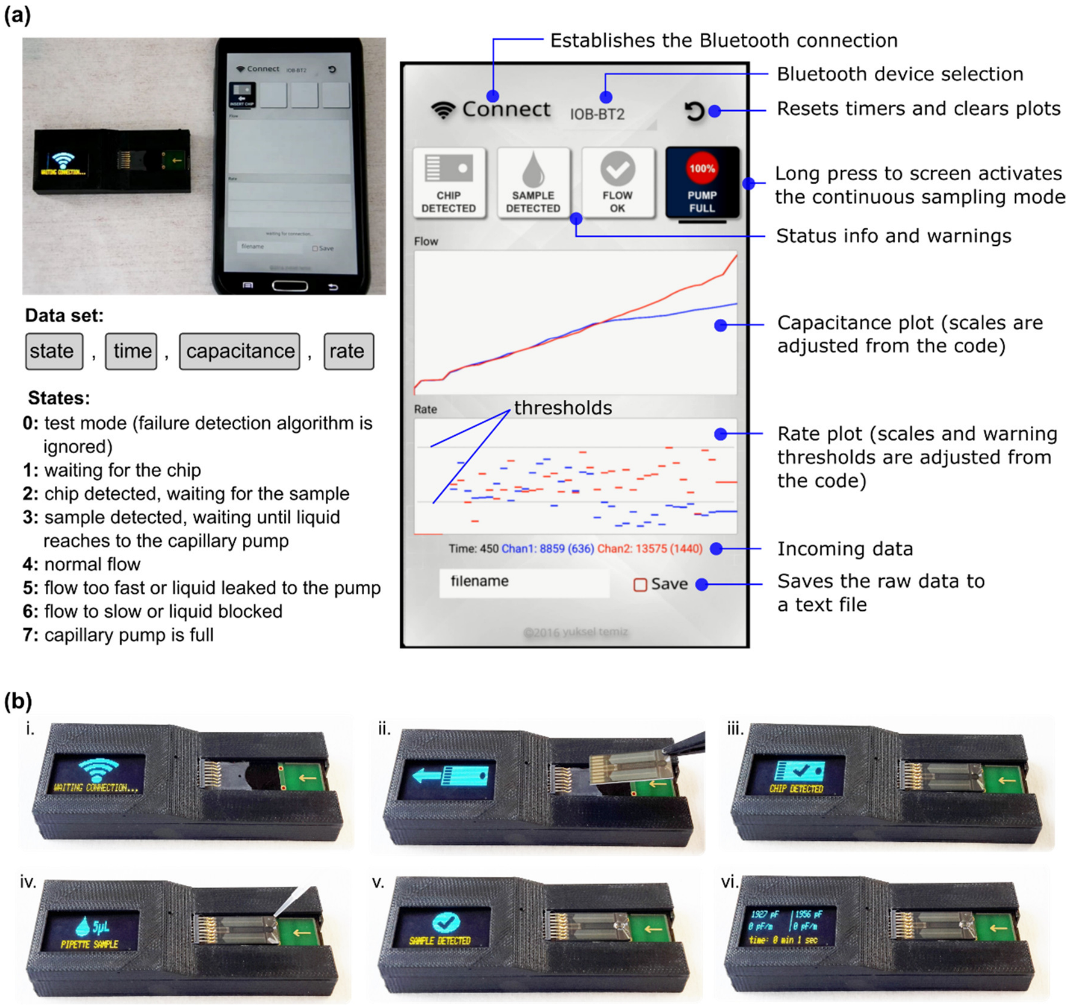

Supplementary Figure S9. Flow monitoring using a portable device and an Android app. (a) Photograph of the portable device and an Android smartphone, and screenshot of the app showing the description of each function. The Android app is written in Java script using the DroidScript (http://droidscript.org/) tool. The app receives the data set via Bluetooth and parses it into different variables. Status and warnings icons are highlighted depending on the "state" parameter. Capacitance, rate, and time parameters for each channel are used to plot the flow information. A "save" option is included to record the raw data to a text file. **(b)** Snapshots from the Device 1 showing major steps from a typical workflow. Images displayed on the OLED display are generated from bitmap images using an image converter software (https://sourceforge.net/projects/lcd-image-converter).
